# Idarucizumab (Praxbind^®^) for dabigatran reversal in patients undergoing heart transplantation: a cohort of ten patients

**DOI:** 10.2144/fsoa-2020-0186

**Published:** 2021-02-15

**Authors:** Eran Kalmanovich, Pascal Battistella, Philippe Rouviere, Bernard Albat, Jean-Marc Frapier, Roland Demaria, Fabien Huet, Audrey Agullo, Marc Mourad, Pascal Colson, Florence Leclercq, Philippe Gaudard, François Roubille

**Affiliations:** 1 Department of Cardiology, Montpellier University Hospital, Montpellier Cedex 5, 34295, France; 2 Department of Cardiovascular Surgery, Montpellier University Hospital, France; 3 Department of Anesthesiology & Critical Care Medicine, Arnaud de Villeneuve Hospital, Montpellier, France; 4 PhyMedExp, Université de Montpellier, INSERM, CNRS, Cardiology Department, CHU de Montpellier, France

**Keywords:** direct oral anticoagulant (DOAC) dabigatran, heart transplantation, idarucizumab

## Abstract

**Background:**

Novel oral anticoagulants are used in atrial fibrillation. Idarucizumab has been approved for reversal of dabigatran in situations of life-threatening hemorrhage or emergency surgery.

**Objectives:**

We report a single center experience of ten patients on dabigatran therapy who were given idarucizumab prior to heart transplantation.

**Methods & results:**

The mean plasma concentration of dabigatran prior to reversal was 139 ± 89 ng/ml. Hemoglobin, hematocrit and platelet levels were decreased after surgery. Surgical procedures were successfully performed with no increased risk, especially regarding bleeding complications. All patients were alive after 90 days.

**Conclusion:**

Dabigatran reversal with idarucizumab in contexts of emergency surgery/urgent procedures is an attractive and safe option to be taken into consideration for patients with end stage heart disease awaiting transplantation and indication of anticoagulant therapy.

## Lay abstract

Novel oral anticoagulants are used in atrial fibrillation. Idarucizumab has been approved for reversal of dabigatran in situations of life-threatening hemorrhage or emergency surgery. We report here a single center experience of ten patients on dabigatran therapy who were given idarucizumab prior to heart transplantation. Surgical procedures were successfully performed with no increased risk, especially regarding bleeding complications. Dabigatran reversal with idarucizumab in contexts of emergency surgery/urgent procedures is an attractive and safe option to be taken into consideration for patients with end stage heart disease awaiting transplantation and indication of anticoagulant therapy.

Atrial fibrillation (AF) is the most common arrhythmia in chronic heart failure (HF) and increases proportionally with the severity of HF, reaching up to 50% in patients with severe HF [1,2]. AF is the main reason for anticoagulant prescription in this population to prevent thromboembolic events [1]. While vitamin K antagonists (VKAs) remain widely used in patients with advanced HF requiring advanced treatments like heart transplantation (HTX), the European Society of Cardiology (ESC) guidelines recommend the use of direct oral anticoagulants (DOACs) over VKAs due to their overall clinical benefit and positive impact on survival, as well as a better safety profile including a reduction of intracranial hemorrhage [3,4] in spite of the residual risk, especially in patients likely to undergo surgery, including HTX [5]. In the context of bleeding events occurring on VKAs or DOACs, blood transfusion and fluid replacement are recommended, with the possible administration of vitamin K for patients on VKAs. However, in severe or life-threatening bleeding events, the management of bleeding is based on the administration of prothrombin complex concentrates or fresh frozen plasma and, when available, a specific antidote [1].

Idarucizumab is a monoclonal antibody fragment developed to reverse the anticoagulant effect of dabigatran specifically [6], an oral thrombin inhibitor indicated in the prevention of stroke or venous thromboembolism in patients with AF [7]. Thus, dabigatran is the only DOAC approved whose anticoagulation action can be specified, rapidly and safely reversed. In cases of not-scheduled emergency procedures, reversion by idarucizumab is expected to prevent hemorrhage-associated complications and to increase the probability of survival during and after life-threatening surgeries like transplantation. This paper reports on a single-center experience including ten patients on dabigatran therapy who were given idarucizumab for emergent reversal before HTX.

## Materials & methods

This cohort study included ten patients suffering from HF who were admitted to the University hospital of Montpellier between October 2017 and December 2018 to undergo HTX. Part of the cohort data have been mentioned recently [8]. In total, there were 21 cases of HTX during this period at our institution. All the patients who were treated with dabigatran and then received 5 g idarucizumab (Praxbind^®^, Boehringer Ingelheim, Paris, France) prior to HTX were to be eligible for the study. Clinical details and laboratory findings at baseline (prior to surgery), during, and 24 h after surgery were obtained from the electronic health record (DxCare software). All values are expressed either as median values with the interquartile ranges or as mean values with standard deviation. The statistics were done using JASP software version 0.9.1.0 [9]. The outcomes were compared with perioperative and postoperative transfusion rates reported in patients who were enlisted for HTX at our center between January 2013 and December 2018, after excluding patients who had a ventricular assist device (temporary or long term) as a bridge to transplantation.

## Results

Patient characteristics are summarized in Supplementary Table. Three out of ten patients had dilated cardiomyopathy as the etiology of HF, and three patients had ischemic cardiomyopathy. The other patients presented with other HF causes, including two cases of restrictive cardiomyopathy (i.e., amyloidosis, sarcoidosis), hypertrophic cardiomyopathy, and one patient had HF due to valvular cardiomyopathy. All patients had an Interagency Registry for Mechanically Assisted Circulatory Support (INTERMACS) score of 3 and above. Two patients had previously undergone thoracotomy, one for valvuloplasty, the other for coronary artery bypass. The median cardiopulmonary bypass (CPB) time was 146.5 ± 39.0 min. The median times of average aortic cross-clamp and on assist after clamp removes were 100.5 ± 13.7 and 25.0 ± 33.0 min, respectively. The mean plasma concentration of dabigatran prior to reversal was 139 ± 89 ng/ml. During CPB, anticoagulation with unfractionated heparin (average of 28,711 ± 9830 UI) was monitored by activated coagulation time (ACT) and was subsequently reversed by using protamine sulfate (average of 11,111 ± 7927 UI) prior to disconnection from CPB. Laboratory results are summarized in Table 1. A marked and significant decrease in hemoglobin levels between presurgery and postsurgery was observed. There was a concomitant decrease in the hematocrit level after surgery. A decrease in platelets was also observed after surgery. Regarding coagulation characteristics, ACT and activated partial thromboplastin time were significantly reduced after surgery compared with with the situation before surgery. Table 2 summarizes the types and number of blood products used during the patients’ hospital stay. Autologous transfusion ranged from 0.3 to 1.1 l, and the number of blood products per patient during their whole hospital stay ranged from 0 to 8. The two patients who underwent a redo surgery (patient number 4, after coronary artery bypass surgery and patient number 5, after mitral valve replacement – see Appendix 1) required more blood products during their stay. They also received a cotreatment with acetylsalicylic acid (ASA), which could explain the excessive bleeding, even though ASA’s presence did not result in excessive bleeding or use of blood in two other cases. In patient number 6, a requirement for more blood products could be explained by hematological dysfunction as part of his sarcoidosis. The median time in intensive care postsurgery was 8.5 ± 10.5 days. Three patients required extracorporeal assist postsurgery while being in the intensive care for up to 72 h, but no graft failure was observed by the end of hospitalization. None of the patients required re-thoracotomy due to bleeding complications, and no thrombotic complications occurred. All patients were alive and well after 30 day follow-up. Five patients completed 90 day follow-up, and four completed 1 year follow-up.

**Table 1.** Laboratory results before, during and after surgery.

	Prior to surgery	During surgery	Postsurgery	Prior vs post surgery p-value	During vs post surgery p-value
Renal function					
– Creatinine (umol/l)	111.5 ± 31.0	NA	135.0 ± 58.2	0.15	NA
– Urea (mmol/l)	10.9 ± 2.65	NA	11.2 ± 3.6	0.14	NA
– GFR	53.5 ± 22.0	NA	57.00 ± 57.2	0.41	NA
– Hematology					
– Hemoglobin (g/dl)	14.15 ± 1.7	9.65 ± 3.0	11.7 ± 1.9	<0.001	0.01
– Hematocrit (%)	41.90 ± 5.6	NA	33.15 ± 5.9	<0.001	NA
– Platelets	272.00 ± 70.7	NA	119.50 ± 48.0	<0.001	NA
Coagulation panel					
– Prothrombin time (s)	12.80 ± 2.0	14.30 ± 0.8	12. 5 ± 1.5	0.26	0.18
– Prothrombin time (%)	87.00 ± 15.0	73.00 ± 5.2	90.50 ± 20.0	0.31	0.12
– Activated partial thromboplastin time (s)	50.2 ± 7.3	73.5 ± 19.3	35.3 ± 5.9	0.16	<0.001
– Activated partial thromboplastin time (ratio)	1.47 ± 0.3	2.27 ± 0.4	1.13 ± 0.1	0.39	0.02
– Fibrinogen (g/l)	2.90 ± 0.9	NA	3.3 ± 0.5	0.49	NA
– Activated coagulation time (s)	140.00 ± 10.5	482.5 ± 39.5	125.5 ± 14.74	<0.001	<0.001
KCT ratio	1.16 ± 0.0	NA	0.96 ± 0.0	0.02	NA
KCTs	33.20 ± 2.0	NA	27.6 ± 3.0	0.02	NA
– Dabigatran level (ng/ml)	139.20 ± 89.03	NA	NA		

KCT: Kaolin clotting time.

**Table 2.** Autologous transfusion and blood products during hospital stay.

Case	Autologous transfusion (l)	PRBCs during	PRBCs after	PLTs during surgery	PLTs after surgery	FFPs during surgery	FFPs after surgery	Other clotting factors	Total blood products during surgery	Total blood products after surgery	Total blood product during hospital
1	0.314	1	0	0	0	0	0	0	1	0	1
2	0.657	0	0	0	0	0	0	0	0	0	0
3	0.898	0	0	0	1	0	0	0	0	1	1
4	0.332	2	2	0	0	0	0	0	2	2	4
5	0.844	2	0	0	0	4	0	2	8	0	8
6	1.128	0	0	0	0	0	0	1	1	0	1
7	0.346	0	0	0	0	0	0	0	0	0	0
8	0.466	0	1	1	0	3	0	0	4	1	5
9	NA	0	0	0	0	0	0	0	0	0	0
10	1.124	0	1	0	0	0	0	1	1	1	2
Mean	0.67 ± 0.31	0.5 ± 0.85	0.4 ± 0.70	0.1 ± 0.32	0.11 ± 0.33	0.70 ± 1.50	0.00	0.4 ± 0.70	1.7 ± 2.50	0.5 ± 0.71	2.20 ± 2.70

FFP: Fresh frozen plasma; PLT: Platelet; PRBC: Packed red blood cell.

**Figure 1.**
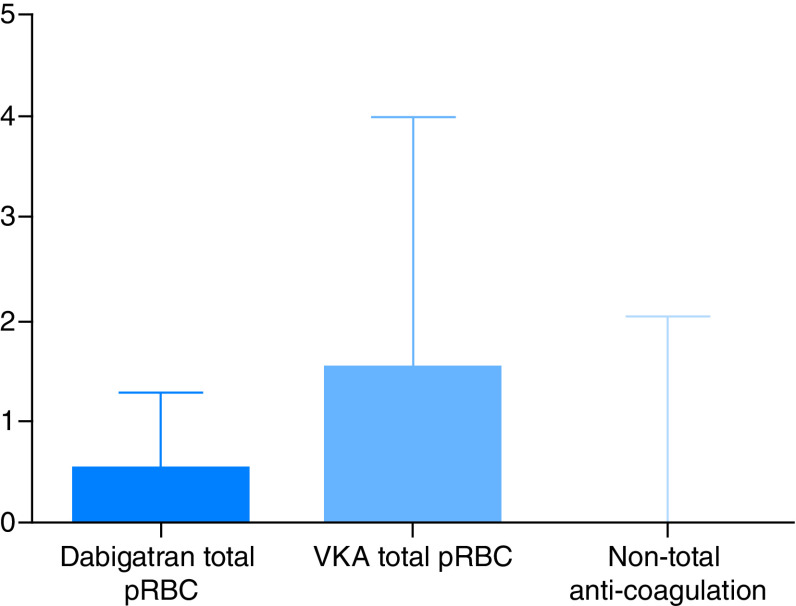
Comparison of packed red blood cell use. Distribution and mean use of pRBC between the different cohort groups. pRBC: Packed red blood cell; VKA: Vitamin K antagonist.

The outcomes were compared with those of patients enlisted for HTX at our center, who had been treated with VKAs (n = 24) or who had not been treated with any anticoagulation before the surgery (n = 21) (Supplementary Table). Patients on the dabigatran group required fewer packed red blood cell (PRBC) units during their perioperative and intensive care unit admission compared with patients treated prior to VKA (0.9 ± 1.30 vs 3.10 ± 4.90; p < 0.01, Figure 1). Similarly, patients in the dabigatran-idarucizumab group required fewer blood products (PRBC, fresh frozen plasma and platelets) in comparison with patients on VKAs during perioperative and postcare (2.20 ± 2.52 vs 8.57 ± 10.56; p < 0.001). There was no difference in the rate of autologous transfusion during the operation between dabigatran and VKA groups (0.67 ± 0.31 vs 0.86 ± 0.41; p = 0.10). No difference was found between the dabigatran group nonanticoagulation groups regarding the rates of either autologous transfusion or blood products consumption during the perioperative and intensive care unit admission.

## Discussion

### Feasibility

This study reports ten successful cases of HTX in patients with AF treated with dabigatran, in which the use of idarucizumab to reverse the effect of dabigatran before surgery allowed positive clinical outcomes with no severe complication. Although the dilute thrombin and ecarin clotting times were not routinely tested in this cohort, plasma concentrations of dabigatran (>30 ng/ml) indicated that all patients were fully anticoagulated prior to surgery, and the routine coagulation panel with prothrombin time (PT), activated partial thromboplastin time (aPTT) and thrombin time (TT) was indicative for the *in vivo* effect of dabigatran. A prolonged aPTT is an indication of an anticoagulation effect of dabigatran, and exhibits a curvilinear relationship with dabigatran concentration with a flattening of the dose-response curve at dabigatran levels greater than 200–300 ng/ml [10]. The current practice of not repeating dabigatran levels after reversal was based on the assumption seen in clinical trials that the 5 g dose of idarucizumab was calculated to reverse the total body load of dabigatran in 99% of patients based on observed dabigatran exposure in the Randomized Evaluation of Long-Term Anticoagulation Therapy trial [11,12].

The first levels of ACT in the operating room were close to the normal values for all patients, which confirmed that the effect of dabigatran had been reversed prior to the procedure [13], making HTX on CPB possible without an excess of bleeding during the procedure and with low use of blood products and coagulation factors. Even though a decrease in hemoglobin levels was observed postsurgery, the concomitant decrease in hematocrit was most likely a reflection of other processes like hemodilution and not of overt bleeding as a cause of hemoglobin decrease. In the postprocedure setting, a low rate of bleeding and the use of blood transfusion were noted, and none of the patients required rethoracotomies due to bleeding complications. Besides, two patients with thoracotomy in their past with higher risk for re-thoracotomies did not experience higher rates of bleeding-related complications.

#### How to choose anticoagulation in patients waiting for HTX

For patients awaiting cardiac transplantation, the choice of anticoagulation therapy is supposed to be based on an optimal anticoagulation efficacy coupled with a low risk of complication, but also the ability to reverse anticoagulation when needed quickly. This aspect is of particular importance given the long waiting period and the inability to predict the availability for transplantation of a donor’s heart. A retrospective cohort of 53 patients having undergone HTX, including 10% of patients on VKAs prior to surgery, highlighted the importance of postoperative bleeding and bleeding complications on the short-term outcome, with a decrease in hemoglobin being a predictive factor of post-HTX blood loss [5]. Data from cohorts of patients on anticoagulation therapy undergoing elective procedures remains limited, and no randomized trials compared VKAs with DOACs in patients undergoing emergent or urgent cardiac surgeries. The Randomized Evaluation of Long-term Anticoagulation Therapy study showed similar rates of perioperative bleeding and thromboembolism in both VKA and dabigatran-treated patients [14]. A retrospective cohort study assessed the rate of bleeding-associated complications in patients on DOACs undergoing elective cardiac surgery (not including HTX) [15]. About 10% of the patients were treated with dabigatran prior to the procedure. The results demonstrated that the DOAC withdrawal period significantly influenced the postoperative 24 h drainage volume. Dabigatran showed a higher drainage volume (p < 0.096), compared with rivaroxaban and apixaban, respectively. Unlike patients treated with rivaroxaban or apixaban, none of the patients treated with dabigatran experienced excessive bleeding requiring re-thoracotomies.

#### Idarucizumab could offer an opportunity for targeted strategy in these patients

The development and recent approval of idarucizumab, a humanized monoclonal antibody fragment specific for dabigatran in patients with life-threatening or uncontrolled bleeding or requiring emergent/urgent procedures, make dabigatran a more convenient anticoagulation option as it can be effectively reversed and then rendered inactive before transplantation or any situation associated with severe bleeding [6,16]. In Phase I/II studies, idarucizumab rapidly and fully reversed dabigatran anticoagulation [17]. The REVERSE-AD study was a prospective open, nonrandomized and noncontrolled study, reporting the management of reversal in 503 patients in which idarucizumab was administered at a dose of 5 g to patients treated with dabigatran and presenting life-threatening hemorrhage (301 patients, group A) or requiring emergency surgery (202 patients, group B) [12]. In group B, periprocedural hemostasis was normal in 184 patients (93.4%), and no patients had severely abnormal hemostasis. Even though hemostasis was normal, many patients had required the use of hemostatic treatment (39.1%), blood transfusions (26.2%) and other blood products, volume expanders, or pro-hemostatic agents (20.8%). Importantly, a subanalysis of the same trial focused on this approach’s interest in patients benefiting from unplanned surgery and confirmed good safety [18]. Notably, the transfusions are less frequent than in our cohort, but surgeries are heterogeneous. Only a few scattered and recent works have reported using idarucizumab to reverse dabigatran before HTX or heart and lung transplantation without an increase in bleeding risk [19–21]. Recently, Jan M. Van Keer *et al.*, published a similar cohort of ten patients enlisted for HTX and treated with dabigatran [22]. The authors reported the benefits of a similar protocol of neutralization of dabigatran with 5 g of intravenous idarucizumab prior to HTX with a complete reversal. The authors did not report an increase in bleeding rates, thrombotic complications, nor interference with heparin use during CPB.

The lack of evidence-based trials likely leads to considerable variabilities in clinical practice. The European Heart Rhythm Association’s guide to DOAC use suggests a general stopping rule of ≥24 h for low-risk procedures and ≥48 h for high-risk surgery. However, longer delays are proposed for patients on dabigatran with a CrCl >80 ml/min or on FXa inhibitors with a CrCl of 15–30 ml/min [3]. In cases of immediate procedures that cannot be delayed (typically cardiac, vascular or neurosurgical emergency procedures), dabigatran reversal with idarucizumab should be considered, especially in moderate-to-high hemorrhagic risk procedures. A recent International consensus statement on peri-operative management of DOACs in cardiac surgery recommends a time-to-interrupt DOACs of 2 days before cardiac surgery, which is always considered as a moderate-to-a high-risk procedure. However, there is no clear cut-off recommendation about the use of idarucizumab in urgent cases [23].

#### Limitations

The study’s main limitation is its design leading to an indirect comparison with patients undergoing HTX while on VKAs. Furthermore, no comparison with other DOACs was possible.

However, the results presented herein show that the use of dabigatran in patients awaiting HTX is feasible and support the use of idarucizumab before the procedure due to its rapid reversal efficacy and its good safety profile regarding the risk of bleeding complications without an increase of thrombotic risk observed with nonspecific reversion. Together with what has been reported from a similar cohort and other evidence in the literature, the results suggest that dabigatran and VKAs are associated with similar rates of periprocedural bleeding, including patients having urgent surgery, but that a better hemostasis control can be achieved pre-operatively with dabigatran after reversion with idarucizumab. Furthermore, the availability of idarucizumab in contexts of emergency surgery/urgent procedures or life-threatening or uncontrolled bleeding has proven to be safe [14].

## Conclusion

The approval and availability of idarucizumab in contexts of emergency surgery/urgent procedures or life-threatening or uncontrolled bleeding is an attractive option to be taken into consideration for patients on dabigatran with end-stage heart disease awaiting transplantation. More clinical trials are needed to confirm these data and better define clinical guidelines.

## Future perspective

Idarucizumab in contexts of emergency surgery/urgent procedures or life-threatening or uncontrolled bleeding is an attractive option to be taken into consideration for patients on dabigatran with end-stage heart disease awaiting transplantation. We present here an appealing option to propose a safe management of these patients. More clinical trials are needed to confirm these data and better define clinical guidelines.

**Summary points**

- Novel oral anticoagulants are used for the prevention of stroke in atrial fibrillation and treatment of thromboembolic events.
- The necessity to reverse their activity in case of urgent procedures with unknown timing, like heart transplantation (HTX), is crucial.
- Idarucizumab has been approved for reversal of dabigatran in situations of life-threatening hemorrhage or emergency surgery.
- We report here a single center experience of ten patients on dabigatran therapy who were given idarucizumab prior to HTX.
- The mean plasma concentration of dabigatran prior to reversal was 139 ± 89 ng/ml.
- Hemoglobin, hematocrit and platelet levels were decreased after surgery, with normalized coagulation panel.
- HTX procedures were successfully performed with no increased risk especially regarding bleeding complications. All patients were alive and well after 90 days.
- Dabigatran reversal with idarucizumab in contexts of emergency surgery/urgent procedures is an attractive and safe option to be taken into consideration for patients with end stage heart disease awaiting transplantation and indication of anticoagulant therapy.
- Before idarucizumab can be used in this setting, whether the dose of plasma dabigatran has to be documented or not, the results have to be confirmed in prospective and large clinical trials.
