# Supplementary material for: Idarucizumab (Praxbind®) for dabigatran reversal in patients undergoing heart transplantation: a cohort of ten patients
Source: Future Sci OA. 2021 Feb 15;7(4):FSO689. doi: 10.2144/fsoa-2020-0186 (PMC8015660; doi:10.2144/fsoa-2020-0186)
Supplement: Supplementary file 1 [file fsoa-07-689-s1.docx]

**Table 1. Baseline characteristics of the patients**

|  | | N=(10 ) | |
| --- | --- | --- | --- |
| Gender | |  |  |
| Male | | 5 (50.0%) | |
| Female | | 5 (50.0%) | |
| Median Age | | 55.5±17.5 years | |
| Type of Cardiomyopathy | |  |  |
| Ischemic | | 3 (30.0%) | |
| Dilated | | 3 (30.0%) | |
| Hypertrophic | | 1 (10%) | |
| Restrictive | | 2 (20.0%) | |
| Sarcoidosis | | 1 | |
| Amyloidosis | | 1 | |
| Valvular | | 1(10.0%) | |
| Comorbidities | |  |  |
| Diabetes melitus | | 0 (0.0%) | |
| Hypertension | | 1 (10.0%) | |
| Dyslipidemia | | 0 (0.0%) | |
| PVD | | 0 (0.0%) | |
| COPD | | 1 (10.0%) | |
| CVA/TIA | | 1 (10.0%) | |
| Active or Past smoking | | 4 (40.0%) | |
| Indication for anticoagulation | | 10(100.0%) | |
| Atrial fibrillation | | 8 (80.0%) | |
| Deep vein thrombosis | | 1 (10.0%) | |
| Left Ventricular apical thrombus | | 1(10.0%) | |
| Medical Treatment | **N=10** | | |
| Beta blockers | 8 (100.0%) | | |
| ACEI | 4 (40.0%) | | |
| MRA | 6 (60.0%) | | |
| Sacubitril/valsartan | 5 (50.0%) | | |
| Aspirin | 4 (40.0%) | | |
| Other antiaggregant | 0 | | |
| Furosemide | 10 (100%) | | |
| Other diuretics | 0 | | |
| Ivabradine | 2 (20.0%) | | |
| Anti-arrhythmic | 3 (30.0%) | | |
| Digoxin | 0 | | |
| Statins | 4 (40.0%) | | |
| Inotropes or vasopressors | 1 (10.0%) | | |

(PVD – peripheral vascular disease; COPD – chronic obstructive lung disease; CVA – cerebrovascular accident; TIA – transient ischemic attack; ACEI – Angiotensin converting enzyme inhibitor; MRA – mineralocorticoids receptors antagonist)
